# Welsh Onion Root (*Allium fistulosum*) Restores Ovarian Functions from Letrozole Induced-Polycystic Ovary Syndrome

**DOI:** 10.3390/nu10101430

**Published:** 2018-10-04

**Authors:** Young Ho Lee, Hyun Yang, Sang R. Lee, Sun Woo Kwon, Eui-Ju Hong, Hye Won Lee

**Affiliations:** 1 College of Veterinary Medicine, Chungnam National University, Daejeon 34134, Korea; lee05@cnu.ac.kr (Y.H.L.) srlee5@naver.com (S.R.L.); ksunwoo12@gmail.com (S.W.K.); 2 Herbal Medicine Research Division, Korea Institute of Oriental Medicine, Daejeon 34054, Korea; hyunyang@kiom.re.kr

**Keywords:** Ovarian failure, Polycystic ovary syndrome, *Allium fistulosum*, LH, FSH

## Abstract

Polycystic ovarian syndrome (PCOS) is an endocrine, metabolic, and systemic disease. It is mainly characterized by hyperandrogenism, oligomenorrhea, and high levels of luteinizing hormone (LH). There is no obvious therapy for PCOS, so patients have received symptomatic therapy. Welsh onion (*Allium fistulosum*) is well-known in Asian countries for its usage in food ingredients and traditional medicines. It is also studied for its many effects. These include activation of immune responses, antihypertensive effects, and antioxidant effects. Using letrozole-induced PCOS rats, we focused on herbal therapy using extract of *Allium fistulosum* (AF; *A. fistulosum*) roots to improve ovarian functions. As a nonsteroidal aromatase inhibitor, letrozole blocks conversion of testosterone to estrogen and subsequently induces PCOS phenomenon. We divided six-week-old female rats into four groups, including control, letrozole, letrozole + AF extract, and temporary letrozole groups. In our study, treatment with AF extract shows a low plasma LH/FSH ratio, and reveals high estrogen levels, ovarian morphology, folliculogenesis-related genes, and aromatase expression under PCOS mimic conditions. We concluded that AF extract administration influenced aromatase production, enhanced the estrogen steroid synthesis, and consequently restored the estrogenic feedback mechanism on the pituitary-ovary system.

## 1. Introduction

Polycystic ovarian syndrome (PCOS) is a common endocrine disorder affecting the health of many women across the world. PCOS patients are usually women of reproductive age suffering from (1) an abnormal estrus cycle, (2) obesity, (3) hirsutism, and/or (4) sub/infertility. Its diagnostic features include hyperandrogenism, ovarian dysfunction, and polycystic ovarian morphology. Although PCOS has no evident cause, it seems that an imbalance among endogenous hormones, especially a high androgen level, as well as insulin resistance, could be considered as possible factors [1,2]. In addition to PCOS, primary ovarian insufficiency (POI) is a common cause of female dysregulation of fertility. POI is a kind of ovarian-related disease which is a characterized by a high level(s) of luteinizing hormone (LH) [3]. Oligo/amenorrhea is the main symptom of POI in women under the age of 40, along with infertility, hot flashes, vaginal atrophy, and dyspareunia [4]. Among the causes of POI, autoimmunity, genetic defect, chemo/radiotherapy, ovarian surgery, and environmental factors have been reported [5]. POI is also characterized by high serum LH and LH/FSH (Follicle-stimulating hormone) ratios [6].

As mentioned above, hyperandrogenism is a key factor in PCOS [7]. In granulosa cells of ovarian follicles, aromatase (cytochrome P450 family 19 subfamily a, Cyp19a1) converts androgen to estrogen. To create an animal model of PCOS similar to human PCOS, we administered letrozole to female rats. Letrozole is a nonsteroidal aromatase inhibitor that suppresses the estrogen biosynthesis in PCOS-like symptoms in rats [8]. Upon hindering aromatase enzyme activity, accumulation of androgen occurred, leading to endocrinal imbalance [8]. We next planted a letrozole pellet to trigger PCOS in female rats, as letrozole blocks aromatase enzyme functions. As a result, ovarian cells cannot produce estrogen, and a polycystic ovary with an abnormal follicular cycle develops due to an increased circulating androgen level [9,10,11]. In our study, we observed weight gain, acyclicity, reduction of estrogen level, hyperandrogenism, and cystic ovarian morphology in letrozole-induced rats.

*Allium fistulosum* (*A. fistulosum*), called Welsh onion, green onion, or spring onion, is a perennial plant that is widely cultivated as cooking material and for traditional medicines in East Asian countries, such as Korea, Japan, and China. As an herbal source of traditional Korean medicine, *A. fistulosum* is used to treat the common cold, febrile disease, abdominal pain, and habitual abortion [12]. Several studies have demonstrated the medicinal effects of *A. fistulosum*, including activation of immune responses [13], inhibition of influenza A virus replication [14], and suppression of obesity, oxidative stress and hypertension [15]. The scaly bulbus of *A. fistulosum* contain essential oils composed of allicin, mucilage, crude fat, crude protein, crude fiber, N-free extract, pentosan, and polysaccharides [12,16].

To this day, there is no definite cure for PCOS. Treatment of PCOS usually depends on the symptoms and focuses on disease management [17]. Although the effects of *A. fistulosum* are not related to female functions, other herbal medicines are commonly used for improvement of body condition [18]. Based on the numerous benefits of *A. fistulosum*, we administered *A. fistulosum* root extract to letrozole-induced rats. In our results, *A. fistulosum* extract treatment relieved hormonal imbalance and altered ovarian function in the letrozole-induced PCOS rat model. Although it is still unknown whether or not *A. fistulosum* extract can effectively treat PCOS patients, our results exhibited the potential restorative effect of *A. fistulosum* extract in letrozole-induced PCOS rats.

## 2. Materials and Methods

### 2.1. Preparation of A. fistulosum (Welsh Onion) Root Extract

*A. fistulosum* (AF) was purchased from a comprehensive oriental medicine company in Naemome DAH (Ulsan, South Korea). Dried AF root (3 kg) was concentrated using a vacuum rotary evaporator and lyophilized using a freeze-dryer. The final AF root water extracts were obtained, amounting to 370.6 g (yield 12.36% *w*/*w*). The plants were deposited in the Herbal Medicine Research Division of Korea Institute of Oriental Medicine (KIOM) in Daejeon, Korea (voucher specimen KIOM M 160074).

### 2.2. Quantitative Analysis of Marker Compounds in A. fistulosum Root Extract

The sample and marker compounds were analyzed by reverse-phase HPLC using a 1100 series high-performance liquid chromatography (HPLC, Agilent Technologies, Santa Clara, CA, USA). The analytical column with a Gemini C18 (4.6 × 250 nm, 5 𝜇m, Phenomenex) was used as the stationary phase and was maintained at 30 °C during the experiment. The mobile phase was composed of distilled water (A) and acetonitrile (B). The gradient flow was as follows: 0–5 min, 5–5% (*v*/*v*) B; 5–10 min, 5–15% (*v*/*v*) B; 10–30 min, 15%–40% (*v*/*v*) B; 30–40 min, 40–40% (*v*/*v*) B; 40–50 min, 40–60% (*v*/*v*) B; 50–60 min, and 60–100% (*v*/*v*) B. The oven temperature was kept constant at 40 °C throughout the analysis. The marker compounds were detected at 254 nm and 330 nm with a flow rate of 1.0 mL/min and injection volume of 10 μL. Identification was based on retention time and UV spectra in comparison with three reference standards: Coumaric acid, ferulic acid, and quercetin. The data were acquired and processed by ChemStation software (Agilent Technologies, Santa Clara, CA, USA).

### 2.3. Animals and Treatments

Female Sprague Dawley rats (6 weeks old, weighing 120–140 g; total *n* = 27, *n* = 6 control group, *n* = 5 temporary letrozole group, *n* = 6 letrozole group, *n* = 10 letrozole + AF extract group) were obtained from Orient Bio Inc. (Seongnam, Korea) and were housed in a pathogen-free facility at Chungnam National University under a standard light/dark cycle of 12:12 h and fed standard chow with water provided ad libitum. Rats were adapted to laboratory conditions for 1 week. All rat experiments were performed in accordance with the Chungnam National University Facility Animal Care Committee standards (approved No. CNU-00849). For the PCOS rat model, a 60-day release letrozole pellet (IRA; Innovative Research of America, Sarasota, OH, USA) (1.8 mg/pellet) was imbedded subcutaneously under anesthesia (isoflurane; Troikaa, Gujarat, India). In detail, rats were placed in an induction chamber and exposed to 2% to 2.5% isoflurane in an O_2_ flowmeter (1 L/min) using an animal anesthesia system (Vetequip^®^, Abesko, Gyeonggi, Korea). After 2 weeks, extract of AF was introduced into the Letrozole + AF extract rat group by per oral (P.O.) treatment (500 mg/kg/body weight) q.d. for 2 weeks, and removal of the letrozole pellet was conducted on Temporary Letrozole group rats after 2 weeks. The AF extract dose was determined according to our previous study [19]. At the end of the experiment, all rats were euthanized with isoflurane overdose.

### 2.4. Serum Hormone Analysis

Blood samples were collected directly from the inferior vena cava with a 1-mL syringe at the end of the experiment. Serum was obtained by centrifugation at 13,000 rpm for 15 min and stored at −70 °C until use. Serum luteinizing hormone (LH) levels were measured using LH ELISA kit (Enzo Life Science, Inc., Farmingdale, NY, USA). Serum follicle-stimulating hormone (FSH) levels were measured using an FSH ELISA kit (Enzo Life Science, Inc., Farmingdale, NY, USA). Serum 17β-Estradiol levels were measured using a 17β-Estradiol ELISA kit (Enzo Life Science, Inc., Farmingdale, NY, USA). Serum testosterone levels were measured using a testosterone ELISA kit (Enzo Life Science, Inc., Farmingdale, NY, USA). All kits were used according to the manufacturer’s instructions, and each sample was analyzed in duplicate.

### 2.5. Reverse Transcription and Real-Time PCR

Total RNA was extracted using TRI Reagent (Molecular Research Center, Cincinnati, OH, USA) following the manufacturer’s instructions. cDNA was synthesized with 1 µg of total RNA with a Thermo Scientific RevertAid First Strand cDNA Synthesis Kit (Thermo, Waltham, MA, USA), and amplified by RT-PCR using AmpliTaq Gold DNA polymerase and Quantitative real-time PCR. cDNA was amplified using Premix Ex Taq (TaKaRa, Shiga, Japan) with SYBR Green I (Molecular Probes, Eugene, OR, USA) by Step One Plus system (Applied Biosystems, Foster City, CA, USA). Primer was synthesized by Macrogen Inc. (Seoul, Korea). Rplp0 expression was used as the control. The primers used for RT-PCR are listed in Table 1. All experiments were run in triplicate, and mRNA values were calculated based on the cycle threshold and monitored for an amplification curve.

### 2.6. Histological Analysis and Immunohistochemistry

Ovary tissue was fixed in 10% buffered formalin for 48 h and was subsequently paraffin-embedded. Paraffin-embedded tissue sections (5 microns) were de-waxed, re-hydrated and stained with haematoxylin and eosin (H&E). The stained slides were examined using a VM600 Digital Slide Scanning System (Motic, CA, USA). Most histological processes were performed at the histological laboratory of the Comparative Animal Resource Center at Chungnam National University. To break protein cross-links, the tissue sections were incubated with a 0.1 M citrate buffer (pH 6.0) at 95–100 °C for 1 h. After blocking with 3% BSA, slides were incubated with Cyp19a1 antibody (1:200 dilution, #14528, Cell Signaling Technology, Beverly, MA, USA) at 4 °C overnight. Following this, slides were washed and incubated simultaneously with corresponding Alexa-Fluor conjugated secondary antibodies from Life Technologies diluted in TBS with 1% BSA at room temperature for 1 h. After washing, slides were mounted in ProLong Gold antifade reagent with DAPI (Life Technologies, Carlsbad, CA, USA) and examined using a DMi8 microscope (Leica Microsystems, Wetzlar, Germany). Vaginal smear was conducted with PBS solution on the week after letrozole pellet imbedding and before sacrifice. Vaginal smear slides were stained with crystal violet (St. Louis, MO, USA) and after washing, slides were mounted in glycerol PBS and examined using a DMi8 microscope (Leica Microsystems, Wetzlar, Germany).

### 2.7. Statistical Analysis

Data are reported as mean ± SEM. Differences between means were obtained by conducting one-way ANOVA followed by Tukey’s multiple comparison test using GraphPad Software (GraphPad Inc., San Diego, CA, USA). *p* < 0.05 was considered statistically significant.

## 3. Results

### 3.1. Quantitative Analysis of Marker Compounds in A. fistulosum Root Extract

**Figure 1:**
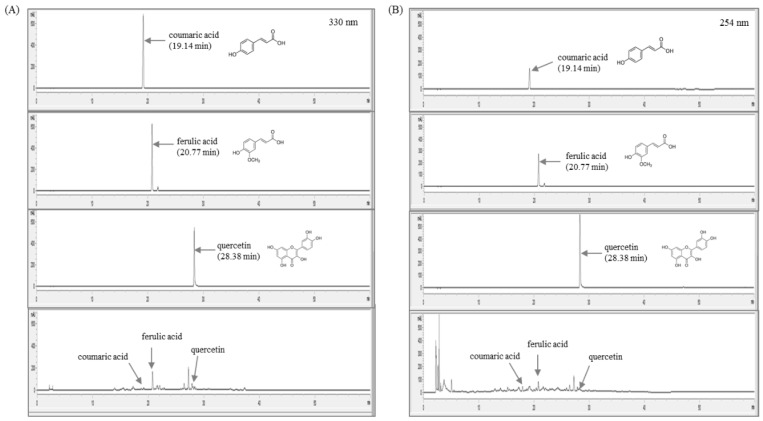
Fingerprinting analysis of *A. fistulosum* root extract and three reference compounds for coumaric acid, ferulic acid, and quercetin at (**A**) 330 nm, and (**B**) 254 nm, respectively.

To identify the chemical constitution and reproducibility of *A. fistulosum*, we performed quantitative analysis using HPLC. As shown in Figure 1A,B, a total of three types of the reference compounds were detected in *A. fistulosum*. Using a stock solution of three reference compounds (coumaric acid, ferulic acid, and quercetin), regression equations were measured for six concentrations in methanol at a concentration of 1.0 mg/mL. Linearity was tested from the correlation coefficient (*r*^2^) of the calibration curves. The linear range, regression equation, correlation coefficients, retention time, and contents of three compounds of the *A. fistulosum* root extract are explained in Table 2.

### 3.2. Animal Condition and Treatments

**Figure 2:**
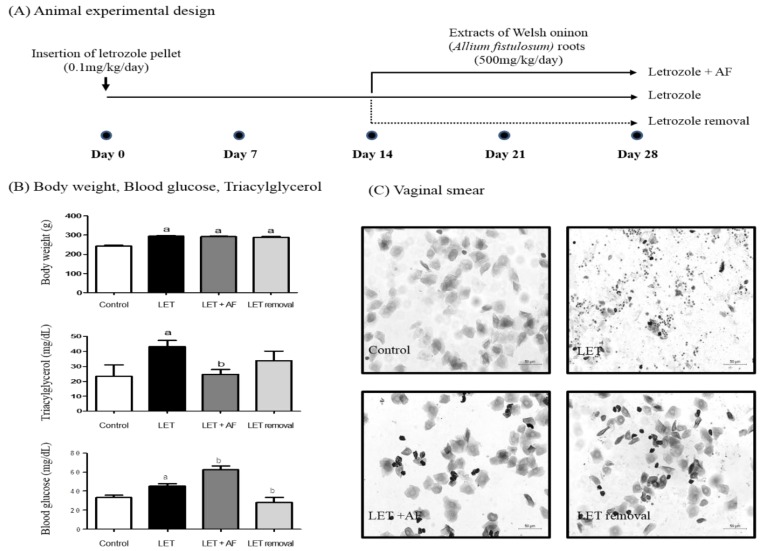
Effect of *A. fistulosum* root extract on physiological changes. (**A**) Animal experimental design. For polycystic ovarian syndrome (PCOS) experiments, a letrozole pellet was imbedded into a six-week-old female rat subcutaneously. On day 14, 2 weeks, *A. fistulosum* extract was treated orally at 500 mg/kg/day for 2 weeks (until day 28) to the LET + AF group, and in the LET removal group, the letrozole pellet was removed at day 14. (**B**) Body weight, triacylglycerol and blood glucose measured at day 28; *n* = 6–10, ^a^ *p* < 0.05 vs. Control group, ^b^ *p* < 0.05 vs. LET group. (**C**) Picture of the vaginal smear performed at the day 28 (200×). AF: *A. fistulosum;* LET: letrozole.

To investigate the improving effect of AF extract treatment on PCOS, we administered AF extract (500 mg/kg/day) for 2 weeks to letrozole-treated rats. For treatment, a letrozole pellet was inserted for 4 weeks (Figure 2A). First, we monitored body weight and the estrus cycle using vaginal smear. Rats were weighed before sacrifice. Body weights of all letrozole-treated groups were heavier than those of the control group (Figure 2B). In letrozole-induced PCOS rats with increased weight gain, glucose, and triacylglycerol, AF extract seemed to help, showing a reduction in blood triacylglycerol when compared to letrozole-induced rats (Figure 2B). Plasma glucose showed an increased level in letrozole-induced rats. While temporary letrozole rats showed reduced glucose levels, treatment with AF extract failed to reduce the blood glucose level (Figure 2B). To assess letrozole-mediated cycle arrest, we monitored the estrus cycle using vaginal smear. As expected, letrozole-induced rats exhibited a prolonged diestrus phase (Figure 2C). Following microscope observation via vaginal smear, the most common cell type was leukocytes in letrozole-treated rats. Interestingly, AF extract-treated rats exhibited epithelial nucleated cells or cornified cells, which indicates restoration of a regular estrus cycle as in control rats. Our findings suggest that AF extract did not affect weight gain caused by letrozole-induced PCOS, whereas it reversed letrozole-induced cycle arrest.

### 3.3. Effect of A. fistulosum Extract on Plasma Hormonal Level

**Figure 3:**
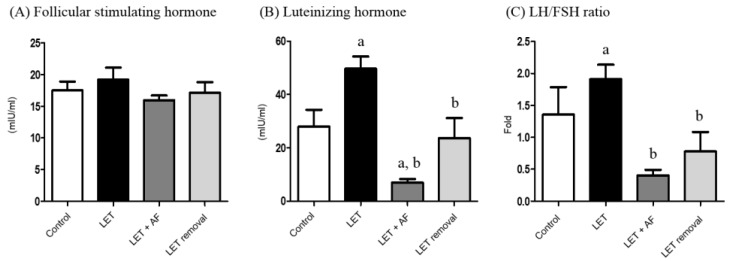
Effect of 2-week treatment with *A. fistulosum* root extract on plasma gonadotropin levels in letrozole-induced PCOS rats. Plasma steroid hormonal levels such as (**A**) FSH, (**B**) LH and (**C**) LH/FHS ratios were measured using a competitive enzyme-linked immunosorbent assay (ELISA) kit. All values represent means ± SEM; *n* = 5–10 per group; ^a^ *p* < 0.05 vs. Control group ^b^ *p* < 0.05 vs. LET group. AF: *A. fistulosum.,* LET: letrozole.

The level(s) of plasma FSH was not significantly altered in all the groups (Figure 3A). Plasma luteinizing hormone (LH) showed an increased level in letrozole-induced rats. However, letrozole + AF extract rats and temporary letrozole rats showed reduced LH levels. Treatment with AF extract significantly reduced the LH level (Figure 3B). Letrozole-induced rats showed an increased LH/FSH ratio, and thus letrozole + AF extract rats and temporary letrozole rats showed decreased LH/FSH ratio levels (*p* < 0.05, vs. Letrozole) (Figure 3C).

### 3.4. Effects of A. fistulosum Extract on mRNA Expression and Histological Changes during Folliculogenesis

**Figure 4:**
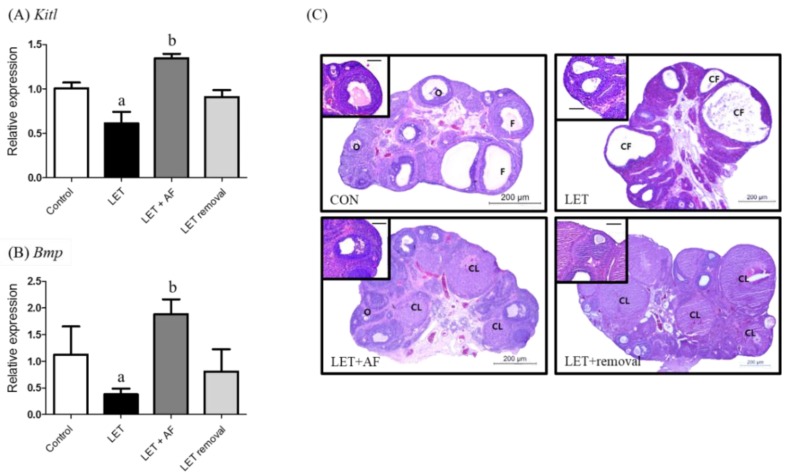
Effects of 2-week treatment with *A. fistulosum* root extract on follicular genesis and growth in letrozole-induced PCOS rats. (**A**) Relative gene expression related to folliculogenesis involving KIT ligand (Kitl) and (**B**) bone morphogenetic protein (Bmp) as assessed by quantitative RT-PCR. (**C**) Section of ovary from each experimental group (H&E, Scale bar = 200 μM, high magnificent scale bar = 50 μM). Rplp was used as in internal control. Values represent means ± SEM; *n* = 5–10 per group; a: *p* < 0.05 vs. Control group; b: *p* < 0.05 vs. LET group. AF: *A. fistulosum*, CF: cystic follicle, CL: corpus luteum, F: normal follicle, LET: letrozole, O: oocyte.

To assess the effect of AF extract on folliculogenesis, the ovarian mRNA level, which is related to follicular genesis of ovarian follicle growth factors such as KIT ligand (Kitl) and bone morphogenetic protein (Bmp), was examined. The Kitl mRNA expression level was reduced in letrozole-induced rats compared to the control and elevated in letrozole + AF extract rats compared to letrozole-induced rats (Figure 4A). Regarding bone morphogenetic protein (Bmp) mRNA expression, letrozole-induced rats showed reduced expression in comparison with the control, whereas letrozole + AF extract rats showed increased expression when compared with letrozole-induced rats (Figure 4B). Formalin-fixed paraffin-embedded rat ovaries were sectioned into 5-µm slices and H&E-stained (Figure 4C). When comparing ovaries between letrozole-induced rats and control rats, the former displayed large subcapsular cysts and a thin granulosa cell layer, while the latter had a more corpus luteum and antral follicles along with a granulosa cell layer. Ovaries of PCOS + AFL and temporary letrozole rats were similar to control rat ovaries in both aspects. The number of follicular cysts was increased in letrozole-induced rats (13.50 ± 4.37) compared to the control (7.83 ± 2.64) and significantly reduced in letrozole + AF extract rats (4.43 ± 1.13) compared to letrozole-induced rats (Table 3).

### 3.5. Effects of A. fistulosum Extract on Hormone Receptor-Related Genes

**Figure 5:**
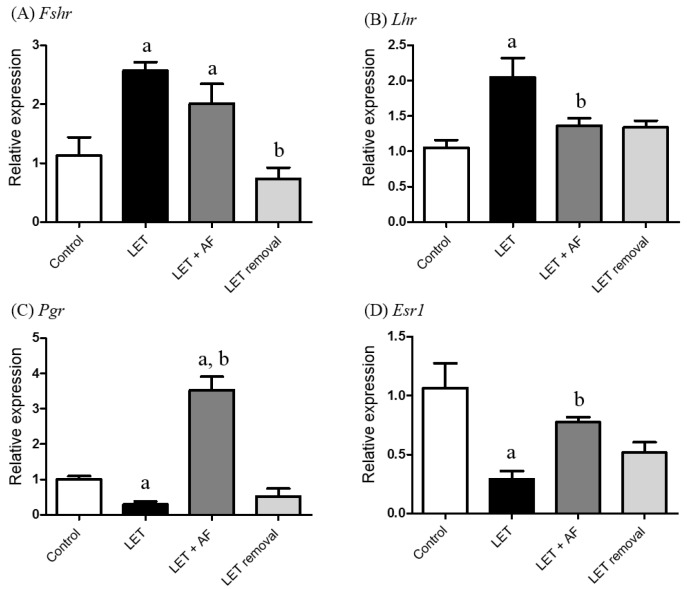
Effect of *A. fistulosum* root extract on ovarian gene expression related to hormone receptors in letrozole-induced PCOS rats. Comparison of mRNA expression levels of (**A**) Fshr, (**B**) Lhr, (**C**) Pgr, and (**D**) Esr1 from ovaries of each experimental group as assessed by quantitative RT-PCR.; Rplp0 was used as in internal control. Values represent means ± SEM; *n* = 5–10 per group; ^a^ *p* < 0.05 vs. Control group ^b^ *p* < 0.05 vs. LET group. AF; *A. fistulosum*; *Esr1: estrogen receptor 1; Fshr*: *follicle-stimulating hormone receptor;* LET; letrozole; *Lhr: luteinizing hormone receptor; Pgr: progesterone receptor.*

To estimate the transcription levels of hormone receptor-related genes, we performed real-time PCR using specific primers for the follicle-stimulating hormone receptor (Fshr), luteinizing hormone receptor (Lhr), progesterone receptor (Pgr), and estrogen receptor 1 (ESR1). In Fshr mRNA expression, PCOS and letrozole + AF extract rats showed increased mRNA expression compared to the control (Figure 5A). The mRNA expression level of Lhr was up-regulated in letrozole-induced rats compared to the control and down-regulated in letrozole + AF extract rats compared to letrozole-induced rats (Figure 5B). The Pgr mRNA level was significantly reduced in letrozole-induced rats, whereas letrozole + AF extract rats showed up-regulated mRNA expression compared to letrozole-induced rats (Figure 5C). Regarding ESR mRNA expression, letrozole-induced rats showed a lower expression level compared to the control, whereas letrozole + AF extract rats showed increased expression compared to letrozole-induced rats (Figure 5D). The results demonstrate that the altered mRNA levels of Lhr, Pgr, and Esr1 by letrozole were restored by AF extract.

### 3.6. Effect of A. fistulosum Extract on Steroid Synthesis-Related Genes

**Figure 6:**
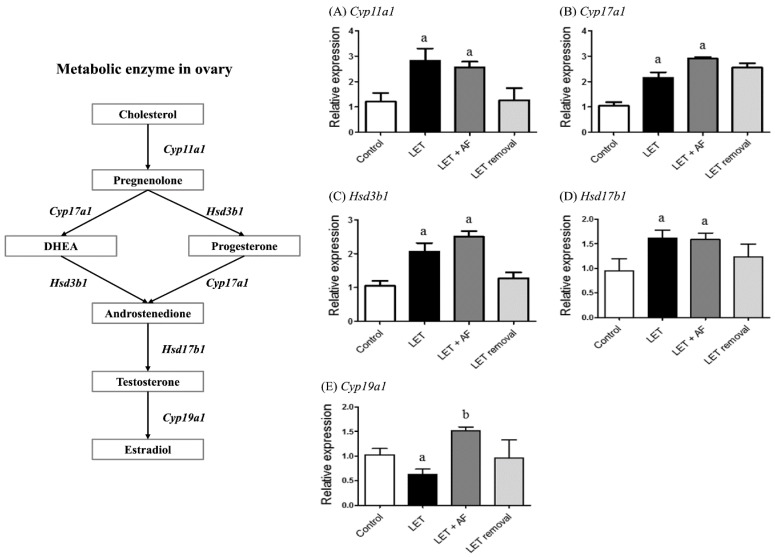
Effect of *A. fistulosum* root extract on ovarian gene expression related to steroid synthesis in letrozole-induced PCOS rats. Comparison of mRNA expression levels of (**A**) Cyp11a1, (**B**) Cyp17a1, (**C**) Hsd3b1, (**D**) Hsd17b1 and (**E**) Cyp19a1 from ovaries of each experimental group as assessed by quantitative RT-PCR.; Rplp0 was used as in internal control. Values represent means ± SEM; *n* = 5–10 per group; ^a^ *p* < 0.05 vs. Control group ^b^ *p* < 0.05 vs. LET group. AF: *A. fistulosum.,* LET; letrozole.

To assess the mRNA expression levels of enzymes related to the steroid biosynthesis pathway, including cytochrome P450, family 17, subfamily a, polypeptide 1 (Cyp17a1), hydroxy-delta-5-steroid dehydrogenase, 3 betahar- and steroid delta-isomerase 1 (Hsd3b1), cytochrome P450, family 11, subfamily a, polypeptide 1 (Cyp11a1), hydroxysteroid (17-beta) dehydrogenase 1 (Hsd17b1), and aromatase (Cyp19a1), we performed real-time PCR. In letrozole-induced rats, higher mRNA expression levels of Cyp11a1, Cyp17a1, Hsd3b1, and Hsd17b1 and lower mRNA expression levels of Cyp19a1 were observed compared with the control (Figure 6). Letrozole + AF extract rats showed increased Cyp19a1 mRNA expression compared to letrozole-induced rats, but there were no differences in Cyp11a1, Cyp17a1, Hsd3b1, and Hsd17b1 mRNA expression compared to letrozole rats. AF extract administration seemed to reverse down-regulation of Cyp19a1 mRNA expression in letrozole-induced rats.

### 3.7. Induction of Aromatase Following Treatment with A. fistulosum Extract Is Linked to Plasma Estradiol Level

**Figure 7:**
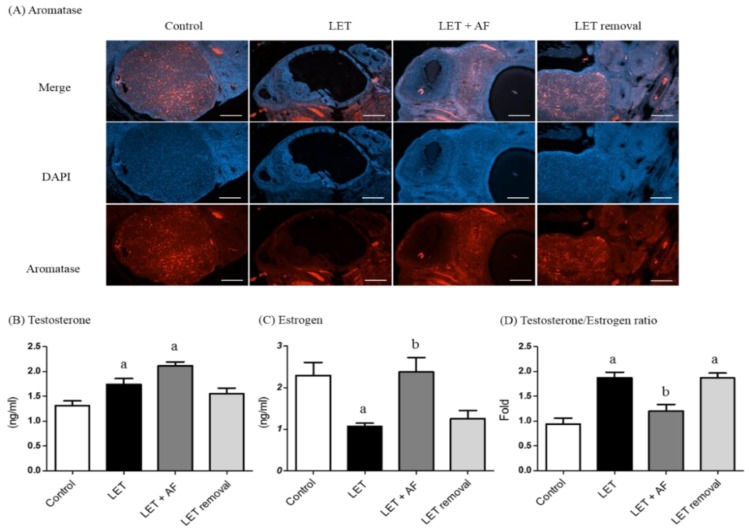
Effect of *A. fistulosum* root extract on ovarian aromatase localization and expression in letrozole-induced PCOS rats. Section of ovary was performed by immunohistochemistry using specific (**A**) aromatase antibody; the 2nd Ab was rabbit (Scale bar = 400 μM), counterstained with DAPI. (**B**) Testosterone and (**C**) estrogen were measured using a competitive enzyme-linked immunosorbent assay (ELISA) kit. (**D**) Testosterone and estrogen ratio. Values represent means ± SEM; *n* = 5–10 per group; ^a^ *p* < 0.05 vs. Control group ^b^ *p* < 0.05 vs. LET group. AF: *A. fistulosum*, LET: letrozole*.*

We performed immunohistochemistry to estimate aromatase expression and localization (Figure 7A). In the ovaries of the control, letrozole + AF extract, and temporary letrozole rats, aromatase was observed mainly in the formed corpus luteum. On the other hand, more follicular cysts were observed in letrozole-induced rat ovaries compared to the control, and less aromatase was detected. Based on these results, AF extract restored ovarian function and improved follicle growth in ovaries of letrozole-induced PCOS rats.

To further investigate the effects of aromatase induction in ovaries, we monitored plasma sex steroid hormones such as testosterone and estradiol. The plasma testosterone level was significantly increased in letrozole-induced and letrozole + AF rats compared to the control (Figure 7B). Plasma estrogen levels were significantly reduced in letrozole-induced rats, whereas letrozole + AF rats showed restoration of estrogen levels similar to control rats (Figure 7C). 

## 4. Discussion

Healthy women of reproductive age experience monthly menstruation after ovulation. This is representative of regular sex steroid production, which is essential for women’s health and systemically affects the development of reproductive organs, endocrinal interactions, and even mentality [3]. Thus, loss of menstruation, except during pregnancy, is a signal indicating ovarian dysfunction. PCOS is a disease affecting many women with endocrine disorders. PCOS is usually observed in climacteric middle-aged women but also occurs in adolescence. PCOS patients show hormonal-related symptoms such as an abnormal estrus cycle, infertility, hirsutism, and obesity [20]. Diagnosis of PCOS is followed by serum hormone analysis and ultrasound scanning of ovaries. These tests can detect high male hormonal levels, ovarian dysfunction, and cystic morphologic changes in PCOS patients. As mentioned, PCOS patients experience hormonal imbalance. High testosterone levels, a high LH/FSH ratio, and low estrogen levels were observed more often in the LET-induced rat group than in the control group through our serum hormonal analysis. In our PCOS animal model, LET-induced rats suppressed ability to synthesize estrogen. Since ovarian hormones were adjusted through a feedback system, lack of estrogen could not handle the FSH/LH ratio. Hormonal disturbance had a negative influence on follicle development, especially failure to progress beyond mid-antral stage, consequently leading to anovulatory infertility. One of the major features of PCOS is a morphological change in the ovary, characterized by lots of follicular cysts. In our ovary section of the LET group, thin-walled and follicular fluid accumulated cyst and absent of corpus luteum could be found. However, AF treated group showed corpus luteum and less follicular cysts. Until now, the cause of PCOS is unknown, which means that doctors can only treat the symptoms. Approximately 50% of women diagnosed with PCOS have coexisting metabolic syndrome, in which insulin resistance is common. Indeed, the risk of developing type 2 diabetes mellitus is five to eight times higher than in women without PCOS [21].

Primary ovarian insufficiency (POI) is ovarian impairment in women younger than 40 years, and it is similar to PCOS in terms of symptoms and ovulation dysfunction. Most POI patients show clinical manifestation, including amenorrhea or oligomenorrhea, infertility, and hypergonadotropic hypogonadism. They are usually treated with exogenous estrogens to prevent complications, followed by hypoestrogenism [22]. POI classification is based on the detection of signaling defects, autoimmunity, insufficient initial follicle number, genomic defects, and chemotherapy [23]. A previous report showed that POI incidence is higher among women with PCOS than in women without PCOS (3.73% vs. 0.44%) [24]. PCOS patients experience interruption of follicle development, resulting in the formation of cysts and eventually follicle depletion. While it is possible that PCOS patients can become naturally pregnant after treatment [25], it is almost impossible for advanced POI patients to conceive naturally.

Chemicals such as androgens (dehydroepiandrosterone (DHEA), testosterone propionate, and 5α-dihydrotestosterone), estradiol valerate, antiprogesterone, and letrozole are used to induce PCOS in mice and rats. These chemicals alter hormonal levels in animals, which develop characteristic features similar to human PCOS, such as hyperandrogenism, high LH levels, and cystic ovarian morphology [26]. Among them, letrozole-induced PCOS rats were the most suitable model for our experiment. Letrozole is a non-steroidal drug that interrupts aromatase, which is a necessary enzyme for estrogen synthesis that converts androgens into estrogens. Thus, we used letrozole-induced PCOS rats as an animal model with similar reproductive and metabolic traits to human PCOS [27]. As shown in another study [9], plasma hormonal levels of letrozole-induced rats showed similar tendencies, including increased LH and testosterone levels and decreased estrogen levels, except for FSH. In addition, the down-regulation of CYP19A1 in letrozole-treated ovaries was consistent with previous evidence showing decreased aromatase expression and estradiol secretion in granulosa cells from DHT-treated rats [28]. Despite its common usage as a medicine, AF is not used for treating PCOS. In this study, we monitored the effects of AF root extract on pathological conditions. There was no significant difference between letrozole-induced and letrozole + AF extract rats in terms of body weight. However, the detection of epithelial nucleated cells or cornified cells from the vaginal smear of letrozole + AF extract rats implies cycle rotation, while letrozole-induced rats showed many leukocytes, implying cycle arrest [29]. Regarding serum hormonal levels, AF extract treatment had good effects on the LH/FSH ratio and serum estrogen levels. FSH and LH play important roles in ovulation, and PCOS patients commonly show a two to three-fold increased LH/FSH ratio, which is sufficient to disrupt ovulation. The serum testosterone level was not affected by AF extract administration, whereas it was elevated in letrozole-induced rats. Hyperandrogenism is a characteristic factor of PCOS. As PCOS is induced by letrozole, which hinders conversion of androgen into estrogen, restoration of the FSH/LH ratio and serum estrogen level implies that AF extract has strong therapeutic efficacy.

Folliculogenesis-related genes, such as Kitl and Bmp, participate in the growth of oocytes by KIT/KITL and the BMP pathway [30]. Transcription of both genes was reduced in letrozole-induced rats and increased in letrozole + AF extract rats. Additionally, in the micrograph of ovarian tissue, letrozole-induced rat ovaries had more cysts than control rats, as well as a thin follicular layer. On the other hand, letrozole + AF extract rat ovaries showed reduction of follicular cysts as well as some corpus luteum. Both results imply the normalization of follicular growth and ovarian cysts by AF extract. The steroid hormone-related receptors rFshb and Pgr showed restoration of mRNA expression after treatment with AF extract. Unlike plasma hormone levels, letrozole + AF extract rats showed greater reduction of Lhr mRNA expression. Regarding steroid synthesis-related gene mRNA expression, the level of Cyp19a1, which is needed to convert androstenedione into estrogen, was restored in letrozole + AF extract rats. Letrozole-induced PCOS rats in other studies as well as ours showed elevation of Cyp17a1, Hsd3b, and Hsd17b1 expression [19,31]. Cyp11a1 acts at the beginning by transforming cholesterol into pregnenolone. Cyp17a1 and Hsd3b1 are needed for conversion of pregnenolone into androstenedione, and Hsd17b1 converts androstenedione into testosterone. These genes were not recovered in letrozole + AF extract rats but were still up-regulated compared to the control. The embedded letrozole pellet acted as an aromatase inhibitor, and restoration of aromatase mRNA expression confirms the positive medicinal effect of AF extract. We also measured protein expression and localization of aromatase using a specific antibody in an IHC experiment in rat ovaries. First, letrozole-induced rat ovaries contained many cysts and showed non-functioning follicles. However, letrozole + AF extract rat ovaries contained less cysts than letrozole-induced rats, more functioning follicles, and more positive signals for proteins.

## 5. Conclusions

All of our experimental results suggest improvement of ovarian function progression upon AF extract treatment to letrozole-induced rats. AF extract promoted aromatase performance, relieved blocked conversion of testosterone into estrogen, increased estrogen, and restored hormonal balance affect ovarian morphology. The effectiveness of AF extract in the recovery of hormonal levels altered by PCOS cannot be underestimated.

## Figures and Tables

**Table 1:** Primers used for real-time or conventional PCR.

Gene Name	Upper Primer (5’–3’)	Lower Primer (3’–5’)
*Kitl*	GGT AGC CAG GAG TTT GTT CT	TTG TGT GGC ATA AGG GCT
*Bmp*	GAT ATT GAG TCT CAG CCC GA	AAC ATG CGG TTG CCT GTA
*Fshr*	CTC ATC AAG CGA CAC CAA GA	GGA AAG GAT TGG CAC AAG AA
*Lhr*	ACA CTG CCC TCC AAA GAA AA	CCT CAA AGA TGG CGG AAT AA
*Pgr*	AGT CTA CCC GCC CTA CCT CA	AGC TCC CAC AGG TAA GCA CA
*Esr1*	GAA GGC TGC AAG GCT TTC TT	TCT TTT CGT ATC CCG CCT TT
*Cyp11a1*	AGG TCC TTC AAT GAG ATC CCT T	TCC CTG TAA ATG GGG CCA TAC
*Cyp17a1*	GCC CAA GTC AAA GAC ACC TAA T	GTA CCC AGG CGA AGA GAA TAG A
*Hsd3b1*	TGG ACA AAG TAT TCC GAC CAG A	GGC ACA CTT GCT TGA ACA CAG
*Hsd17b1*	ACT TGG CTG TTC GCC TAG C	GAG GGC ATC CTT GAG TCC TG
*Cyp19a1*	ATG TTC TTG GAA ATG CTG AAC CC	AGG ACC TGG TAT TGA AGA CGA G
*Rplp0*	CTC AGT GCC TCA CTC CAT CA	CTT CCT TTG CTT CGA CCT TG

**Table 2:** Linearity, retention time of three reference compounds, and the contents of *A. fistulosum* root extract.

Compound	Detection Wavelength (nm)	Retention Time (min)	Linear Range (μCg/mL)	Regression Equation (y = ax + b)	Correlation Coefficient (*r*^2^)	Content ^†^ (μg/mg)
Courmaric acid	330	19.14	3.0–200	y = 29.462x − 141.067	0.999	0.168 ± 0.005
Ferulic acid	330	20.77	3.0–180	y = 28.045x − 107.988	0.999	0.988 ± 0.005
Quercetin	254	28.38	3.0–200	y = 34.2023x − 154.1267	0.999	2.591 ± 0.037

^†^ Content was express as mean ± standard deviations.

**Table 3:** Number of follicular cysts.

Group	CON	LET	LET + AF	LET Removal
Number	7.83 ± 2.64	13.50 ± 4.37	4.43 ± 1.13 *	3.60 ± 0.55 *

* *p* < 0.05 vs. LET group.
